# Chromosomal constitutions of five wheat – *Elytrigia elongata* partial amphiploids as revealed by GISH, multicolor GISH and FISH

**DOI:** 10.3897/CompCytogen.v11i3.11883

**Published:** 2017-08-03

**Authors:** Fang He, Yuhai Wang, Yinguang Bao, Yingxue Ma, Xin Wang, Xingfeng Li, Honggang Wang

**Affiliations:** 1 State Key Laboratory of Crop Biology, Shandong Key Laboratory of Crop Biology, College of Agronomy, Shandong Agricultural University, Taian 271018, People’s Republic of China; 2 Zaozhuang University, Zaozhuang 277160, People’s Republic of China

**Keywords:** *Elytrigia elongata*, partial amphiploid, cytogenetic, *in situ* hybridization

## Abstract

A combination of meiotic pairing analysis and *in situ* hybridization (genomic *in situ* hybridization [GISH], multicolor GISH [mcGISH] and fluorescence *in situ* hybridization [FISH]) of five *Triticum aestivum* (Linnaeus, 1753) - *Elytrigia elongata* (Podpěra, 1902) (2n = 10x = 70) amphiploids was employed to investigate the genomic constitution and relationships between wheat and alien chromosomes. GISH, multicolor GISH and FISH patterns of mitotic chromosomes indicate that the genomic constitution of the five partial amphiploids (XY693, XY7430, SN19, SN20 and SN122) are 14A + 12B + 14D + 8Js + 8J, 12A + 16B + 14D + 2St + 8Js + 2J + 2 W-E, 14A + 14B + 14D + 4St + 8Js, 14A + 14B + 14D + 2St + 10Js + 2J, and 14A + 14B + 14D + 2St + 8Js + 4J, respectively. Analysis of meiotic chromosome pairing in the F_1_ hybrids between these five partial amphiploids suggests that SN20 and SN122 are the most closely related amphiploids and are somewhat related with XY693 and XY7430. However, the alien chromosome constitutions of SN19 differed from the other four amphiploids. In addition, a new pairing between wheat and *E. elongata* chromosomes was distinguished in some cells of the hybrids SN19 × XY7430, SN20 × XY7430 and SN122 × XY7430.

## Introduction

*Elytrigia* (Á. Löve, 1980) species, such as those in the Triticeae tribe of the family Poaceae, contain excellent perennial forages with resistance to cold, drought, salinity, and various diseases (Mao et al. 2010). Among these, *E. elongata* was determined to have many agronomically useful traits for wheat improvement, such as a high protein content (Garg et al. 2009); tolerance to salt (Zhong and Dvorak 1995) and drought (Roundy 1985); resistance to stripe rust (Yang and Ren 2001), leaf rust (Friebe et al. 1996), *Fusarium* head blight (Scoles et al. 2009; Shen et al. 2004) and wheat streak mosaic virus (Li and Wang 2009). These genes have been transferred into wheat and extensively used to improve resistance to various pests and tolerance to abiotic stresses.

Previous research indicated that *E. elongata* is an autodecaploid with the JJJJJ genomes proposed by Muramatsu (1990). Further probing experiments using the E genome for blocking and the St genome as a probe revealed that the genomic composition of *E. elongata* is JJJJsJs (Chen et al. 1998c). The J genome of *E. elongata* is homologous to the J genome of the diploid *Thinopyrum bessarabicum* (Săvulescu & Rayss, 1923) and harbors St signals in the telomeric regions, whereas the Js genome is a modified J genome of unknown origin characterized by the presence of a St genome-specific hybridization signal near the centromere. Thus, the St genome probe (plus the genomic DNA of common wheat, with or without the E genome DNA as a block) could be used as a diagnostic tool to distinguish the St-, Js-, and J-genome chromosomes from the wheat chromosomes in wheat - alien amphiploids.

Wheat - alien amphiploids, which are stable and highly fertile, represent a crucial intermediate step in the transfer of agronomically useful genes from wheatgrass species to wheat (Fedak et al. 2000; Fedak and Han 2005; Jiang et al. 1994). Over recent decades, several wheat - *E. elongata* amphiploids have been developed and identified as promising sources of multiple disease resistance in various laboratories throughout the world (Chen et al. 1998a; He et al. 2013; Li et al. 2005; Oliver et al. 2006; Sepsi et al. 2008b; Zhang et al. 1996b). These amphiploids, which contain complete genomic sets of wheat plus a set of alien chromosomes, were developed by backcrossing the wheat and *E. elongata* hybrids to wheat. Thus, they provide useful intermediate material for studying the genomic affinities among alien chromosomes. These amphiploids are also useful for producing wheat - alien addition, substitution and translocation lines, which possess biotic stress resistance not found in common wheat.

Developed approximately 30 years ago in China, XY693 and XY7430 are two wheat - *E. elongata* partial amphiploids with 56 chromosomes. They possess many useful genes. XY693 exhibits excellent resistance to leaf rust, powdery mildew and Wheat streak mosaic virus (Li et al. 2005). Three partial amphiploids (SN19, SN20 and SN122) were derived from the cross *T. aestivum* ‘Shannongfu63’// *T. aestivum* ‘Yannong15’/ *E. elongata* and exhibit resistance to powdery mildew (He et al. 2013) and stripe rust (F. He and H. G. Wang, unpublished data). Being stable and highly fertile, genetic material of these amphiploids could serve as a potential source for wheat improvement. Li et al (2003) and He et al (2013) reported that the alien genome composition of XY693 (Li et al. 2003), SN20 and SN122 (He et al. 2013). However, the wheat genome compositions of these amphiploids were ignored. Zheng et al. (2014) examined the chromosome compositions of XY693 and XY7430 using GISH, FISH and mcGISH. However, a few investigations have focused on the homoeologous and compensatory relationships among *E. elongata* and wheat chromosomes between these wheat - *E. elongata* amphiploids.

Genomic *in situ* hybridization (GISH) is a widely used and effective method for detecting alien chromatin in wheat-alien species amphiploids. Multicolor GISH (mcGISH), which employs several different genomic probes, can be used to simultaneously visualize two or more genomes in a polyploid species. Han et al. (2004) used mcGISH to analyze wheat - *Thinopyrum intermedium* (Host, 1805) derivatives and detected intergenomic rearrangements involving *Th. intermedium* chromosomes and the A and B genomes of wheat. Fu et al. (2012) successfully used mcGISH to differentiate the A, B, D and E genomes of wheat - *E. elongata* addition, substitution and translocation lines. Fluorescence *in situ* hybridization (FISH), which uses repetitive DNA clones, is a powerful tool for identifying chromosomes within a species (Gill et al. 1993; Komuro et al. 2013; Pedersen and Langridge 1997) or tracing intergenomic chromosome rearrangements in a polyploid species (Danilova et al. 2014). The combination of the mcGISH technique with sequential FISH on the wheat alien hybrids enables chromosomes belonging to different genomes to be detected and identified, such that intergenomic rearrangements within a polyploid species can be visualized (Nagy et al. 2002; Wang et al. 2005).

In the present study, we used a combination of GISH, mcGISH and FISH to examine the cytogenetic composition of five partial amphiploids. In addition, the relationships between wheat and the alien chromosomes of the wheat - *E. elongata* amphiploids were assessed via the combination of the meiotic behavior of the F_1_ hybrids.

## Material and methods

### Plant materials

Plant materials used in this study included *E. elongata*, *Pseudoroegneria spicata* (Pursh, 1814) (StSt, 2n = 14), *Triticum urartu* (Gandilyan, 1972) (AA, 2n = 14), *Aegilops speltoides* (Gerlach & Dyer, 1980) (SS, 2n = 14), *Aegilops tauschii* (Cosson, 1850) (DD, 2n = 14), the common wheat Yannong15 and five partial amphiploids (XY693, XY7430, SN19, SN20 and SN122). *E. elongata*, *T. urartu*, *A. speltoides* and *A. tauschii* were provided by Prof. Zhensheng Li (formerly of the Northwest Institute of Botany at the Chinese Academy of Sciences, Yangling, China). *Ps. spicata* was provided by Prof. Lihui Li (Institute of Crop Science, Chinese Academy of Agricultural Sciences, Beijing, China). XY693 was derived from the cross *T. aestivum* ‘Xiao Yan 2’// *T. aestivum* ‘Lin 7’/ *E. elongata*. Similarly, XY7430 was derived from the cross *T. aestivum* ‘Xiang Yang 4’// *T. aestivum* ‘mi sui zao’/ *E. elongata*. The partial amphiploids SN19, SN20 and SN122 were novel germplasms developed from the hybridization of *E. elongata*, the common wheat Yannong15 and Shannongfu63 at the Agronomy College of Shandong Agricultural University, Tai’an, China. All plant materials are maintained in our laboratory through self-crosses.

### Genomic *in situ* hybridization (GISH)

Seeds were germinated at 25°C on moist filter paper in Petri dishes, maintained at 4°C for approximately 24 h, and then transferred to 25°C. Roots 1- to 2-cm in length were cut and treated in ice water for approximately 24 h before fixation in Carnoy’s solution. After fixation, the root tips were stained and squashed in carbol fuchsin, and their mitotic chromosomes were observed under a microscope. When the plants reached the flag leaf stage, spikes were sampled and anthers at metaphase I (MI) of meiosis were fixed in Carnoy’s solution, dissociated in 1 M HCl at 60°C for 6 to 8 min, and homogenized in 1% acetocarmine. *E. elongata* and *Ps. spicata* DNA were labeled with fluorescein-12-dUTP by the nick translation method and used as probes. Sheared genomic DNA from YN15 (AABBDD, 2n = 42) was used as blocking DNA. The slides were counterstained with propidium iodide (PI, 0.25 mg/mL) in the Vectashield mounting medium (Vector Laboratories, USA).

### Multicolor genomic *in situ* hybridization (mcGISH)

The total genomic DNA was isolated from young leaves of *E. elongata*, *T. urartu*, *A. speltoides* and *A. tauschii*. The total genomic DNA of diploid *E. elongata* and *T. urartu* was labeled with fluorescein-12-dUTP, and the total genomic DNA of *A. tauschii* was labeled with Texas-red-5-dUCP via the nick translation method. The total genomic DNA of *A. speltoides* was used for blocking (at a ratio of 1:160). After hybridization, the slides were washed in 2 × saline sodium citrate (SSC) and mounted in Vectashield mounting medium (containing 1.5 mg/mL DAPI; Vector Laboratories, USA).

### Fluorescence *in situ* hybridization (FISH)

FISH was performed after mcGISH analysis using two probes, pTa535 labeled with fluorescein-12-dUTP, and pSc119.2 labeled with Texas-red-5-dUCP. The two probes were mixed at a ratio of 1:1 before hybridization. The slides were washed in 2 × SSC and mounted in Vectashield mounting medium (containing 1.5 mg/mL DAPI; Vector Laboratories, USA). The detailed procedures for the chromosome preparation and hybridization were previously described by Han et al. (2004). Photographs were captured with an Olympus BX-60 fluorescence microscope equipped with a CCD (charge-coupled device) camera.

## Results

### McGISH analysis of five partial amphiploids

To further determine the five partial amphiploids by mcGISH, the mitotic chromosomes were probed using D-genomic DNA (*A. tauschii*) with Texas-red-5-dCTP (red), and the total genomic DNA of *E. elongata* and *T. urartu* was probed with fluorescein-12-dUTP (green) and blocked by the S-genome (*A. speltoides*) DNA. Using this technique, the A-, B-, and D-genomes from common wheat and the genomes from *E. elongata* could be simultaneously distinguished. The A-, B-, and D-genome chromosomes were labeled with yellow, brown/ gray and red/ pink fluorescence, respectively, whereas the alien chromosomes of *E. elongata* were labeled with green fluorescence.

**Figure 1.**
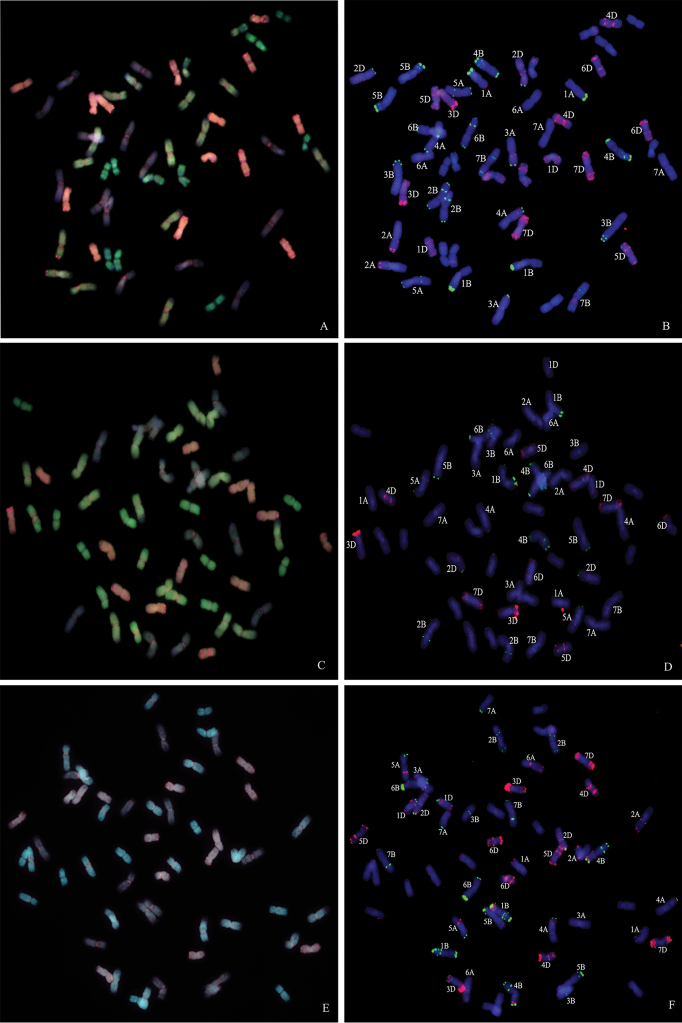
McGISH patterns and FISH analysis of chromosomes inSN19 (**A**), SN20 (**C**) and SN122 (**E**). Yellow denotes the A-genome chromosomes, gray indicates the B-genome chromosomes, red represents the D-genome chromosomes and green denotes the *E. elongata* chromosomes or chromosomal fragments. The asterisks indicate the wheat - *E. elongata* translocation chromosomes. FISH on the same metaphase chromosome spreads are simultaneously presented in lines SN19 (**B**), SN20 (**D**) and SN122 (**F**) by pTa535 (green) and pSc119.2 (red).

Chromosome constitutions of these five wheat - *E. elongata* amphiploids (XY693, XY7430, SN19, SN20 and SN122) were analyzed using mcGISH (Table 1). As illustrated in Fig. 4A, XY693 contained 16 alien chromosomes plus 40 wheat chromosomes, including 14 A-genome, 12 B-genome, and 14 D-genome chromosomes. XY7430 had 12 green-fluorescing chromosomes that originated from *E. elongata* and two interspecific translocation chromosomes with green terminal fragments on red short arms. Of the remaining 42 wheat chromosomes, 12 fluoresced yellow, 16 gray, and 14 red. Therefore, in addition to the 12 alien chromosomes and 2 wheat - *E. elongata* translocation chromosomes (Fig. 4B, asterisk), XY7430 also possessed 12 A-genome, 16 B-genome, and 14 D-genome chromosomes (Fig. 4B). Similarly, SN19 possessed 12 *E. elongata* chromosomes and 42 wheat chromosomes, including 14 A-genome, 14 B-genome, and 12 D-genome chromosomes (Fig. 1A). Both SN20 and SN122 carried 14 *E. elongata* chromosomes plus 42 wheat chromosomes, including 14 A-genome, 14 B-genome, and 14 D-genome chromosomes (Fig. 1C, E).

**Table 1.** GISH analysis of five partial amphiploids.

Line	Chromosome no.	Origin of alien chromosomes	Genomic chromosome constitution*
XY693	2n=56	16 of *E. elongata*	14A + 12B + 14D + 8Js + 8J
XY7430	2n=56	12 of *E. elongata*	12A + 16B + 14D + 2St + 8Js + 2J + 2 W-E
SN19	2n=54	12 of *E. elongata*	14A + 14B + 14D + 4St + 8Js
SN20	2n=56	14 of *E. elongata*	14A + 14B + 14D + 2St + 10Js + 2J
SN122	2n=56	14 of *E. elongata*	14A + 14B + 14D + 2St + 8Js + 4J

* W-E, wheat - *E. elongata* translocation chromosomes

### FISH analysis of five partial amphiploids

Although the mcGISH patterns of the mitotic chromosomes indicated that the wheat - *E. elongata* amphiploid SN19 has 54 chromosomes and the remaining four partial amphiploids have 56 chromosomes, the number of alien chromosomes ranged from 12 to 16, suggesting that chromosome deletion and substitution occurred in the wheat genome. Three-color FISH, with the simultaneous hybridization of the repetitive DNA probes pTa535 and pSc119.2, has been successfully employed on mitotic metaphase cells of these five partial amphiploids. Chromosome elimination and addition has been detected with these probes (Figs 4C, Dm 1B, D, F). The results of the mcGISH analysis revealed that a pair of B-genome chromosomes was absent from XY693 and a pair of B-genome chromosomes was added from XY7430 (Fig. 4A, B). Comparing their FISH results using probes pTa535 and pSc119.2 with those for common wheat, the missing and added chromosomes were determined to be 6B and 2B, respectively. In addition, the FISH results revealed that the wheat - *E. elongata* translocation chromosomes in XY7430 were wheat chromosome 5D with a terminal alien segment on the short arm.

**Figure 2.**
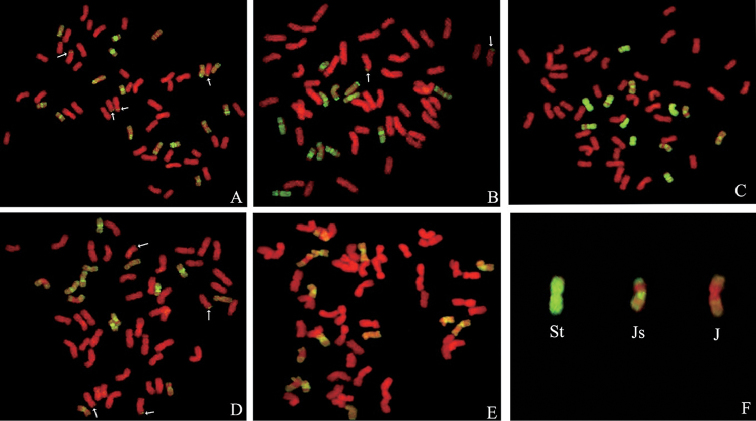
GISH patterns of the mitotic chromosomes probed with St-genomic DNA from *Ps. spicata* and blocked with ABD-genomic DNA from Yannong15 wheat. **A** XY693 **B** XY7430 **C** SN19 **D** SN20 **E** SN122 **F** Karyotype of chromosomes from Fig. 1C organized into three types. Arrows indicate translocated chromosomes.

**Table 2.** Average metaphase I configuration per meiocyte in the F_1_ hybrids between partial amphiploids.

Hybrid	No. of plants	No. of cells	Meiotic configuration of amphiploids hybrids chromosomes					No. of alien chromosomes	Meiotic configuration of *E. elongata* chromosomes		
			I	II_ring_	II_rod_	III	IV		I	II_ring_	III_rod_
SN20 × SN19	6	83	11.81 (7-21)	17.54 (16-20)	3.88 (1-6)	0.04 (0-1)	0.06 (0-1)	13	9.1 (7-13)	1.03 (0-2)	0.92 (0-1)
SN19 × SN122	5	64	12.08 (9-17)	16.59 (16-18)	4.75 (2-6)	0	0.06 (0-1)	13	9.45 (5-13)	0.99 (0-3)	0.78 (0-1)
SN122 × SN20	5	79	3.44 (2-6)	21.49 (17-24)	4.78 (3-8)	0.01 (0-1)	0	14	2.5 (2-4)	3.96 (3-4)	1.79 (1-2)
SN19 × XY7430	5	66	10.93 (7-18)	16.37 (14-19)	5.51 (2-9)	0.05 (0-1)	0.04 (0-1)	12	9.32 (7-15)	0.77 (0-5)	0.57 (0-1)
SN20 × XY7430	6	76	5.98 (4-12)	17.2 (12-19)	6.37 (4-9)	0.11 (0-1)	0.64 (0-3)	13	4.18 (1-11)	3.06 (1-5)	1.35 (0-3)
SN122 × XY7430	7	88	8.38 (4-14)	15.7 (13-20)	7.29 (3-9)	0.27 (0-1)	0.21 (0-1)	13	6.1 (1-11)	2.76 (1-4)	0.69 (0-2)
XY693 × SN122	6	94	5.98 (2-12)	17.14 (16-19)	7.68 (7-9)	0.13 (0-1)	0	15	5.26 (3-11)	2.94 (1-4)	1.93 (0-3)
XY693 × SN19	7	69	11.61 (5-13)	15.96 (15-17)	5.05 (5-8)	0.14 (0-1)	0.24 (0-2)	14	10.24 (2-12)	0.87 (0-3)	1.01 (0-3)
XY693 × SN20	6	85	6.11 (2-10)	17.34 (15-19)	6.92 (4-9)	0.14 (0-1)	0.24 (0-2)	15	5.68 (3-9)	2.84 (2-4)	1.82 (1-3)

**Figure 3.**
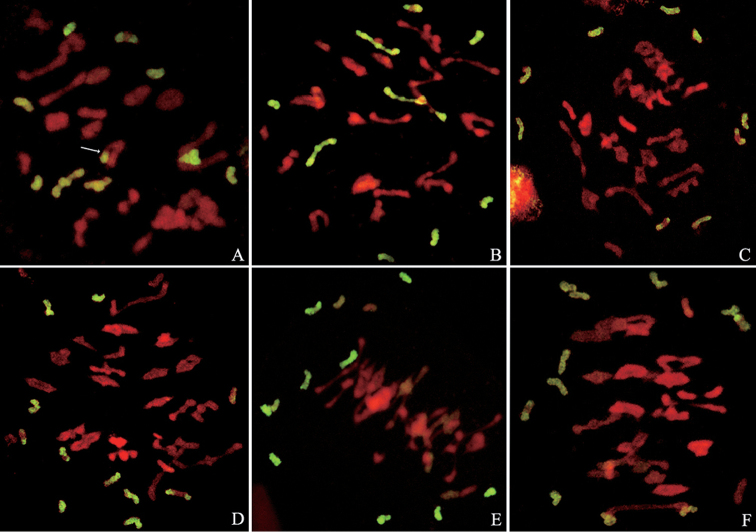
GISH patterns of meiotic chromosomes probed with *E. elongata* genomic DNA and blocked with Yannong15 wheat genomic DNA. **A** SN122 × XY7430 **B** SN20 × XY7430 **C** XY693 × SN20 **D** XY693 × SN122 **E** SN20 × SN19 **F** SN19 × SN122. Arrows indicate pairing between the wheat and *E. elongata* chromosomes.

**Figure 4.**
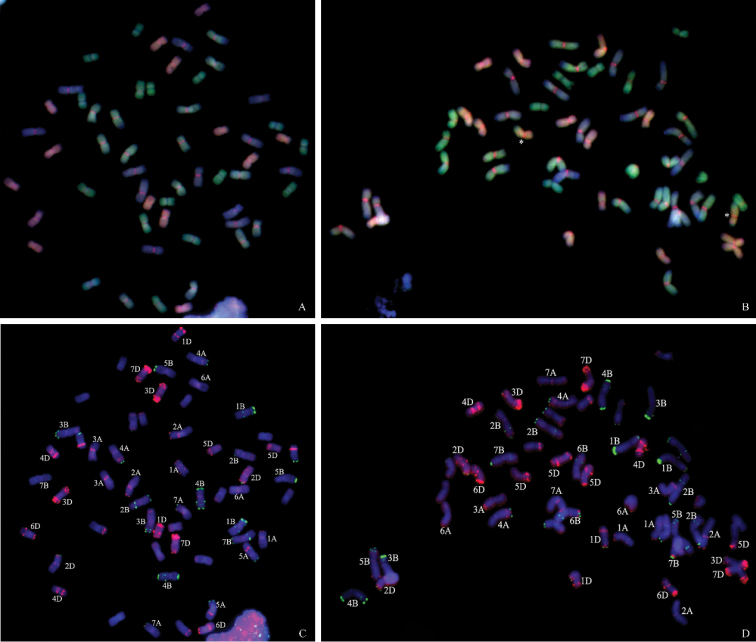
McGISH patterns and FISH analysis of chromosomes in XY693 (**A**), XY7430 (**B**). Yellow denotes the A-genome chromosomes, gray indicates the B-genome chromosomes, red represents the D-genome chromosomes and green denotes the *E. elongata* chromosomes or chromosomal fragments. The asterisks indicate the wheat - *E. elongata* translocation chromosomes. FISH on the same metaphase chromosome spreads are simultaneously presented in lines XY693 (**C**), XY7430 (**D**), by pTa535 (green) and pSc119.2 (red).

### GISH identification

The alien chromosome constitutions of five wheat - *E. elongata* amphiploids were analyzed using GISH (Table 1). The assignment of the alien chromosomes present on partial amphiploids were based on the differences in the type of signal obtained with wheat DNA blocking and St genome probing (Chen et al. 1998b) (Fig. 2F).

Probing the partial amphiploid XY693 with St genomic DNA and blocking with wheat DNA revealed that 16 chromosomes emitted greenish-yellow hybridization signals: a Js type of signal was detected on 4 pairs of chromosomes, a J type of signal on 8 chromosomes, and four translocation signals on the telomere of four wheat chromosomes (Fig. 2A). The partial amphiploid XY7430 had 12 chromosomes from *E. elongata*, including 2 St-genome chromosomes, 8 Js-genome chromosomes, 2 J-genome chromosomes, and 2 wheat - *E. elongata* translocated chromosomes (Fig. 2B). The results with SN20 were similar to those with XY693 and XY7430, except that the SN20 contained 2St-, 10 Js- and 2 J-genome chromosomes as well as 4 wheat - *E. elongata* translocated chromosomes (Fig. 2D). In addition, the partial amphiploids SN19 and SN122 did not contain wheat - *E. elongata* translocated chromosomes. The genomic constitution of these two alien chromosomes was 4 St + 8 Js (Fig. 2C) and 2 St + 8 Js + 4 J (Fig. 2E), respectively.

### Meiotic analysis of the partial amphiploids

Five partial wheat - *E. elongata* amphiploids were crossed with each other, and their meiotic chromosome behavior was studied in all nine F_1_ hybrids (except XY693 × XY7430). *E. elongata* chromatin was distinguished in the pollen mother cells (PMCs) at MI of the F_1_ hybrids using GISH, and the values for each meiotic parameter are presented in Table 2.

As presented in Table 2, most of the nine F_1_ hybrids exhibited high frequencies of univalents and low frequencies of multivalents, including trivalents and quadrivalents. All F_1_ hybrids exhibited high frequencies of pairing between the wheat chromosomes, whereas the multivalents were absent in the *E. elongata* chromosomes, and univalents were observed for both the wheat and *E. elongata* chromosomes. In addition, pairing chromosomes between wheat and *E. elongata* were observed in some cells of the hybrids SN19 × XY7430, SN20 × XY7430 and SN122 × XY7430 (Fig. 3A, arrows). The F_1_ hybrid between XY693 and XY7430 was not involved in this experiment considering that the configuration of these amphiploid hybrids and the relationship of the alien chromosomes between XY693 and XY7430 were analyzed in earlier studies.

In contrast to the regular meiotic behavior of the parents, the values for the F_1_ hybrids were highly variable. Based on the lowest frequencies of unpaired chromosomes, the most closely related strains were SN20 and SN122. Only an average of 2.5 *E. elongata* chromosome univalents per cell was observed in the hybrid SN122 × SN20. Another grouping involved SN20 × XY7430 (Fig. 3B), SN122 × XY7430, XY693 × SN20 (Fig. 3C) and XY693 × SN122 (Fig. 3D). In this grouping, an average of 4.18 and 6.1 *E. elongata* chromosome univalents was observed in the hybrids of XY7430 with SN20 and SN122, respectively. Also, the F_1_ hybrids of XY693 with SN20 and SN122 exhibited 5.26 and 5.68 *E. elongata* chromosome univalents, respectively. The strain SN19 appeared to be unrelated to the other partial amphiploids, as indicated by the high frequency of unpaired chromosomes in the hybrids. In addition, 9.1, 9.45, 9.32 and 10.24 unpaired *E. elongata* chromosomes were observed in the F_1_ hybrids obtained from crossing SN19 with SN20, SN122, XY7430 and XY693, respectively (Fig. 3E, F). In addition, a new pairing between the wheat and *E. elongata* chromosomes was distinguished in some cells of the hybrids SN19 × XY7430, SN20 × XY7430 and SN122 × XY7430 (Fig. 3A, arrows).

## Discussion

To date, numerous studies have reported that many of the chromosomes contained in alien genomes are products of translocations either between *Elytrigia* chromosomes or with wheat chromosomes. Using GISH and mcGISH, Han et al. (2004) demonstrated that the wheat - *Th. intermedium* amphiploid Zhong 1 carried a translocation between a pair of A genome chromosomes and *Th. intermedium* chromosomes and that another wheat - *Th. intermedium* amphiploid Zhong 4 contained rearrangements between the A and D genomes of wheat. Similar results were obtained by Sepsi et al. (2008a), who found 4A and 7A chromosome pairs carrying telomeric translocations using mcGISH and FISH on the wheat - *E. elongata* amphiploid BE-1. Oliver et al. (2006) proved that wheat - *E. elongata* amphiploid SS363 carries 14 *E. elongata* chromosomes, 40 wheat chromosomes, and two wheat *E. elongata* translocated chromosomes. The wheat - *E. elongata* partial amphiploids XY693 and XY7430 were developed approximately 30 years ago in China. Li et al. (2003) distinguished eight Js genomes and eight J genome chromosomes in line XY693. Zheng et al (2014) determined that the chromosome compositions of XY693 and XY7430 using GISH, FISH and mcGISH. In this study, we report similar results for XY693 but distinguished two pairs of translocated chromosomes. In addition, one pair of wheat - *E. elongata* translocated chromosomes in XY7430 and 4 translocated chromosomes in SN20 were also detected.

The genomic composition of the decaploid species *E. elongata* has been a subject of interest for a considerable time (Chen et al. 1998c; Liu and Wang 1993; Zhang et al. 1996a). When a somatic chromosome preparation from this species is blocked with E genomic DNA and probed with St-genomic DNA, a “centromeric signal” was observed on 28 chromosomes, faint telomeric signals were observed on 42 chromosomes, and uniform St signals were not noted in any genome. Thus, the genomic constitution of this species was designated as JJJJsJs (Chen et al. 1998c). In contrast, Zhang et al. (1996a) concluded that the St genome is also present in *E. elongata* and that the centromeric region is critical in distinguishing these chromosomes from E- (=Js-) or J-type chromosomes, which are also present in this species. In the current study, four of these five partial amphiploids exhibited one or two pairs of St-genomic chromosomes. This result indicates that the basic genomes of *E. elongata* were J, Js and St. The presence of the major discrepancy in the genome symbols may be explained by the varied origins of *E. elongata*. However, additional research is needed to confirm this hypothesis.

Chromosome counting on the metaphase spreads after GISH revealed 16 alien chromosomes in the partial amphiploid XY693 and 12 alien chromosomes in the partial amphiploid XY7430. However, the chromosome number of these two amphiploids was consistently 56. This observation suggests that the chromosome substitutions occurred between the *E. elongata* genome and wheat genome. Similar results were obtained in previous studies. Fedak et al. (2000) reported the genomic composition of six wheat - *E. elongata* amphiploids (PMW706, PMW206, PMW209, PMWIII, OK7211542 and an *Agropyron* - wheat hybrid) revealed by GISH using St-genomic DNA as a probe. The number of alien chromosomes varied from 12 to 18 among the studied amphiploids. Similarly, using the St genomic DNA as a probe, Sepsi et al. (2008a) demonstrated the genomic composition of a wheat - *E. elongata* amphiploid BE-1 included 16 alien chromosomes and 40 wheat chromosomes. FISH analysis identified the substituted wheat chromosome pair as 7D. Afterwards, other wheat - *E. elongata* amphiploids, such as Agrotana, ORRPX and SS660, were also found to contain 40 wheat and 16 alien chromosomes when applying GISH, but the substituted wheat chromosomes were not identified (Chen 2005; Fedak and Han 2005; Oliver et al. 2006). This observation suggested that in the preserved process of these amphiploids, a wheat chromosome substituted an alien chromosome and then duplicated spontaneously. However, the homologous group of the chromosomes involved in the substitution has not been identified. Further research on the internal mechanism for this spontaneous substitution would thus be helpful in understanding the genetic relationship among the genomes of *S. cereale*, *L. mollis* and wheat as well as in the development of alien substitution lines.

Zhang et al. (1995) implied that meiotic pairing control genes (*ph* genes) present in the decaploid species *E. elongata* genomes based on the homoeologous chromosome pairing were strongly promoted in such hybrids. The *ph* genes might be present in parts of the wheat - *E. elongata* amphiploids. However, no direct evidence is available to prove this deduction. Oliver et al. (2006) analyzed the chromosome pairing configurations of six F_1_ hybrids from four partial wheat - *E. elongata* amphiploids hybridized with each other. Unfortunately, no pairing between the wheat and *E. elongata* chromosomes was observed in any of these hybrids. In this study, a new pairing between wheat and *E. elongata* chromosomes was distinguished in some cells of the hybrids SN19 × XY7430, SN20 × XY7430 and SN122 × XY7430. These results demonstrated that the *E. elongata* chromosomes in partial amphiploids XY7430 contain *ph* genes.

The data from the GISH and meiotic chromosome pairing analysis in the F_1_ hybrids between different partial amphiploids were useful in identifying similar alien genomes. Based on the lowest frequency of *E. elongata* unpaired chromosomes, the two strains SN20 and SN122 would appear to be the most closely related of all the partial amphiploids examined. The highest frequency of the univalent *E. elongata* chromosome in the hybrids SN20 × SN19, SN19 × SN122, XY693 × SN19 and SN19 × XY7430 suggested that the alien chromosome constitutions of SN19 differed from the other four amphiploids. High *E. elongata* chromosome univalent and bivalent frequencies were observed in the other hybrids, including SN20 × XY7430, SN122 × XY7430, XY693 × SN122 and XY693 × SN20. These results indicate that the partial amphiploids SN20 and SN122 were related to XY693 and XY7430. Previous reports demonstrated that the alien chromosome constitutions of XY693 and XY7430 differed (Zhang and Dong 1994). Generally, these five partial amphiploids originated from the same alien parent and do not necessarily carry the same combination of alien chromosomes and the alien genomes in partial amphiploids, which are quite complex. The synthetic nature of the alien genomes in the partial amphiploids has been reported by several authors (Chen 2005; Chen et al. 1998b; Fedak and Han 2005; Sepsi et al. 2008a). These partial amphiploids are cytogenetically stable and highly fertile, perhaps because they are homologous and there is compensation between the *E. elongata* chromosomes and wheat chromosomes.

Partial wheat – *E. elongata* amphiploids, with a high cross-compatibility with wheat, are desirable ‘‘bridge’’ materials for transferring disease resistance genes from *E. elongata* to wheat. In previous studies, these five partial amphiploids demonstrated regular meiotic behavior and are highly fertile. Based on the chromosome constitution of these amphiploids, we manipulated *Elytrigia* chromosomes in these amphiploids to eliminate unwanted *Elytrigia* chromatin and to introduce useful agronomic traits into wheat. Due to the unpredictable and unstable meiotic behavior of the F_1_ hybrids between the lines, the combining of traits by intercrossing partial amphiploids is not a promising alternative.
